# A Metabolic Perspective and Opportunities in Pharmacologically Important Safflower

**DOI:** 10.3390/metabo10060253

**Published:** 2020-06-17

**Authors:** Vimalraj Mani, Seon-Kyeong Lee, Yunsoo Yeo, Bum-Soo Hahn

**Affiliations:** 1 Department of Agricultural Biotechnology, National Institute of Agricultural Sciences, Rural Development Administration, Jeonju 54874, Korea; vimalraj08@gmail.com (V.M.); lsk220@korea.kr (S.-K.L.); ysyeo@korea.kr (Y.Y.); 2 National Agrobiodiversity Center, National Institute of Agricultural Sciences, RDA, Jeonju 54874, Korea

**Keywords:** safflower, crop, metabolites, function, pharmacological compounds

## Abstract

Safflower (*Carthamus tinctorius* L.) has long been grown as a crop due to its commercial utility as oil, animal feed, and pharmacologically significant secondary metabolites. The integration of omics approaches, including genomics, transcriptomics, metabolomics, and proteomics datasets, has provided more comprehensive knowledge of the chemical composition of crop plants for multiple applications. Knowledge of a metabolome of plant is crucial to optimize the evolution of crop traits, improve crop yields and quality, and ensure nutritional and health factors that provide the opportunity to produce functional food or feedstuffs. Safflower contains numerous chemical components that possess many pharmacological activities including central nervous, cardiac, vascular, anticoagulant, reproductive, gastrointestinal, antioxidant, hypolipidemic, and metabolic activities, providing many other human health benefits. In addition to classical metabolite studies, this review focuses on several metabolite-based working techniques and updates to provide a summary of the current medical applications of safflower.

## 1. Introduction

**Figure 1:**
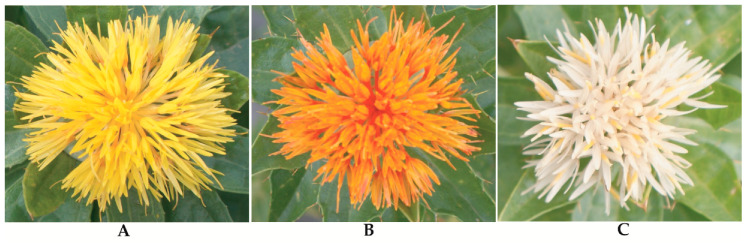
Differential flowering phenotypes of *Carthamus tinctorius* L.: (**A**) Yellow (The Germplasm Resources Information Network database, PI251398), (**B**) red (PI253529), and (**C**) white (PI209297).

**Figure 2:**
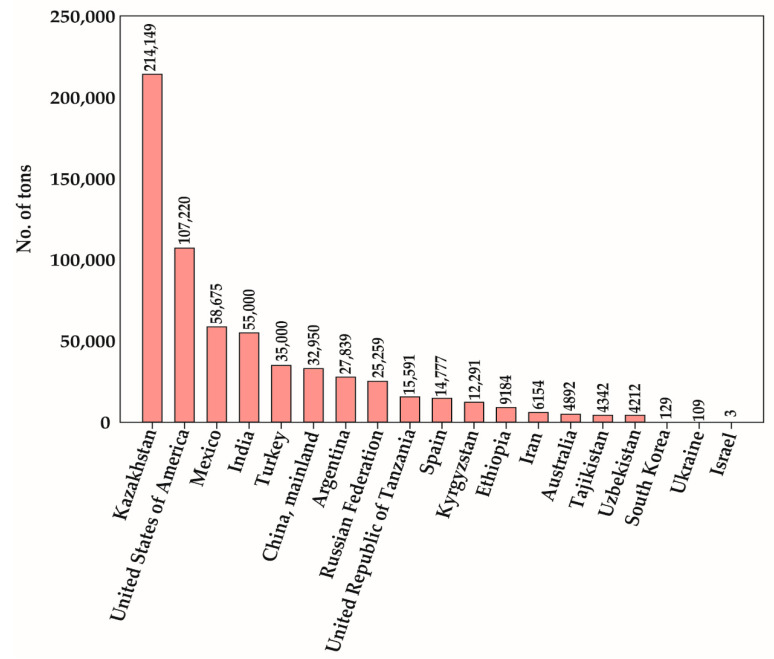
List of world’s top safflower-producing countries in 2018. The total number of tons of production value for each country is shown in the graph. The country-wise production statistics were obtained from FAOSTAT (2020) [9], while the production value of South Korea was obtained from the database of Ministry of Agriculture, Food and Rural Affairs, Korea.

The genus *Carthamus* is a diverse group of plants within the family *Compositae*, or *Asteraceae*, comprising 15 species of east Mediterranean origin. Most of the species are diploid, but there are three polyploidy species (*Carthamus creticus*, L., *Carthamus lanatus* L., and *Carthamus turkestanicus* Popov) [1]. Safflower, considered relatively drought- and salt-tolerant compared to other oil seed crops, is suitable for cultivation in dry areas [2]. The crop has mainly been cultivated for its flower and oil content in seeds. Safflowers are also used as edible cooking oil, food coloring, fabric dyes, animal and birdfeed, medicines, and biofuel production [3,4]. The major composition of fatty acid in safflower seed oil determines its commercial uses [5]. Based on the differential varieties, flowers usually display in yellow, orange, red, and white colors [6] (Figure 1). Safflower is known for its flowers, which were traditionally used for coloring and flavoring in the food industry. Safflower was cultivated before a large production of cheaper aniline dyes became available for textile-staining dyes [7]. Due to its special medicinal characteristics, safflower seeds and flowers are often harvested via handpicking [8]. More than 60 countries grow safflower for multiple purposes. Among all these countries, Kazakhstan, USA, Mexico, India, Turkey, and China are the highest producers of safflower [9] (Figure 2). Due to high demands for its oil, safflower cultivation has increased to compensate for the lack of nutritional oil [10]. Safflower has two major types, spiny or spineless. Spiny safflower has a higher oil content than the spineless safflower plants. Until now, Asian countries have mainly delivered the demand for safflower florets; moreover, the existing cultivation is not enough to meet the increasing demand. Therefore, cultivation guidelines must be established for other regions. Furthermore, the selection of cultivars, as well as their origins, is crucial factors which finally determines flower color, as well as the number of capitula and branches [11]. Safflower contains some of the healthiest oils for human consumption. However, it is still the minor crop worldwide. Hence, the main breeding goals are to increase the yield, seed oil content, and disease resistance [12]. 

Omics is a recent approach used to address the universal detection of genes (genomics), mRNAs (transcriptomics), proteins (proteomics), and metabolites (metabolomics) in a specific biological sample, having a broad range of applications [13]. To obtain a large amount of omics data, various high-throughput technologies are used. A current trend involves combining omics using a platform to manage the large dataset to determine the biological source. Safflower is mostly a self-pollinating dicot crop with an estimated haploid genome size of 1.4 GB. This crop suffers from a lack of well-developed genetic resources [14]. A minimum of 285 nucleotide sequences and 41,000 expressed sequence tags (ESTs) were reported as available for safflower, which were identified through the subtractive genomic library and composite database. In 2006, a dedicated genomic resource for safflower, SemBioSys, was developed. This contains the safflower bacterial artificial chromosome (BAC) library, seed expressed sequence tag (EST) library, seed-specific promoters, and protein coding genes of lipid metabolic pathways such as oleosin and other seed storage protein genes [15]. This has enabled robust genetic developments toward improving agronomic traits in safflowers. This genomic resource helps breeders to obtain new cultivars much faster and easier than using conventional breeding techniques. In an attempt to identify flowering genes in safflower, Liu et al. employed 454 pyrosequencing and identified at least 51,591 unigenes via de novo assembly. The 51,591 unigenes were mapped to 281 Kyoto Encyclopedia of Genes and Genomes (KEGG) pathways and classified into 43 main functional groups. These data provided a foundation for further studies on secondary metabolism in safflower [16].

Due to its traditionally known pharmacological importance, the metabolites and chemical profile of safflower, its synthesis, and diversity have been exploited by plant breeders, biochemists, and pharmacologists. Several attempts have been made to decode its distinct metabolic profiles through high-throughput metabolic fingerprinting methods, nuclear magnetic resonance (NMR), liquid chromatography (LC-MS), and gas chromatography (GC-MS), so-called metabolomics [17]. These methods have allowed us to provide a review of safflower metabolomics and its wide range of applications in the medicine and food industries. The metabolic analyses of the chemical components of safflower other than pigments have revealed the presence of organic acids, fatty acids, polyphenolic compounds, phytosterols, free sugars, and minerals [18]. Over 200 chemical compounds have been isolated from safflower (*Carthamus tinctorius*), and the most commonly known are flavonoids, coumarins, fatty acids, and polysaccharides [19]. Thin layer chromatography (TLC) and high-performance liquid chromatography (HPLC) are the two most widely used techniques for qualitative metabolomics in safflower. Seed phenolic compounds, such as *N*-feruloylserotonin-5-*O*-*β*-d-glucoside, 8-hydroxyarctigenin-4-*O*-β-d-glucoside, leutolin-7-*O*-*β*-*d* glucoside, and *N*-feruloylserotonin, were found to be predominant and can be quantified using HPLC. Based on their ubiquitous presence, hydroxysafflor yellow A (HSYA) and kaempferide were chosen as two markers to determine the quality of safflower. Chakradhari et al. recently profiled both lipophilic and hydrophilic components of cultivated *C. tinctorius* as well as wild *C. oxyacantha* safflower using liquid chromatography-high resolution mass spectrometry (LC-HRMS/MS) [20]. Therefore, the objective of this present review is to emphasize the pharmacologically important extract or metabolic compounds of safflower seeds and flowers for their possible potential implications in medicine.

## 2. Botanical and Morphological Characteristics

Safflower is a member of the *Asteraceae* family which constitutes approximately 22,750 genera and more than 1620 species in the order *Asterales*. *Carthamus* species may originate from Southern Asia, and are annual thistle-like plants with many spines on leaves and bracts, cultivated mainly in dry, hot climate conditions [21]. They can reach a height of 0.3 to 2.1 m and their axillary flowers grow in the leaf axils. Flowers are initially orange and later change into a red color. The total bloom stage may last for four weeks or more. The heads with upper leaves are up to 4 by 3 cm long. The bracts are light green and have thorny tips with a thorny appendage. The fruit is 6 to 8 cm long, obovate or pear-shaped, and bluntly wedge-shaped at the base with protruding long ribs. The species of *Carthamus* has a thin fusiform root and its stem is erect, simple, or branched at the top into stiff, glabrous, whitish-yellow, and glossy branches. The leaves are long, rather soft, and glabrous with a thorny-serrate margin and tip. The size of the leaf varies widely from species to individual plant and usually ranges from 2.5 to 5 in width and 10 to 15 cm in length [22]. 

## 3. Safflower Chemical Composition

### 3.1. Lipophilic Compounds 

#### 3.1.1. Fatty Acids 

Oilseeds are some of the major sources of vegetable oils used primarily for nutritional, industrial. or pharmaceutical applications, as determined by their fatty acid composition. The fatty acid composition is highly variable depending on the species of the plant and the environmental effects [23]. Numerous studies were reported on the influence of environmental factors such as salinity reduction related to the fatty acid composition and/or the yield of essential oil [24]. The ratio of the seed oil (oleic/linoleic acid) was shown to greatly depend on the temperature and humidity factors during seed maturation [25]. Indian safflower cultivars (ISF1, ISF2, and ISF3) were shown to produce more oleic acid level under irrigated conditions than in dry climatic conditions [26]. Besides external factors, various internal factors also determine the fatty acid profiles and plant growth under different environmental conditions, and geographical locations had characteristic differences in fatty acid compositions and contents [27,28]. Safflower oil contains two main unsaturated fatty acids: Oleic (18:1) and linoleic acid (18:2), which compose 90% of the total fatty acids. The remaining 10% includes saturated fatty acids like palmitic (16:0) and stearic acid (18:0). Standard safflower oil contains about 6–8% palmitic acid, 2–3% stearic acid, 16–20% oleic acid, and 71–75% linoleic acid [29]. However, in many other studies, the fatty acid composition of safflower seeds showed considerable variability [30]. Early studies detailed the fatty acid compositions of 200 safflower accessions originating from 37 countries, indicating that oleic and linoleic acid have a tremendous range of variation from 3.1% to 90.60% and from 3.9% to 88.8%, respectively [31]. The fatty acid compositions of oil from cultivated and wild germplasms do not show significant differences, indicating that the seeds of wild safflower can be effectively used for human consumption and industrial purposes. The oil content was 29.20–34.00%, 20.04–30.80% and 15.30–20.80% in *C. tinctorius*, *C. oxyacantha* Bieb., and *C. lanatus* L., respectively [32]. Due to the high content of unsaturated fatty acid, safflower has been widely used as a cooking oil in countries such as India, USA, Mexico, Spain, and Australia [33]. To date, 14 fatty acid components have been successfully analyzed using HPLC for safflower seeds [34].

#### 3.1.2. Tocopherols

The vitamin E (α-, β-, γ-, and δ-tocopherol) profile was identified in safflower germplasm [35]. Recently, Velasco and Fernández–Martínez reported that γ-tocopherol constitutes approximately 10% of the total tocopherols in *C. tinctorius* [36], whereas, the natural mutant of *C. oxyacantha* has >90% γ-tocopherol in seed, which is more than the standard high α-tocopherol content usually found in wild-type seeds. As the mutant showed introgression of *C. tinctorius*, simultaneous selection for high γ-tocopherol content and morphological traits produced a high γ-tocopherol safflower line, designated IASC-1 [37]. Recent studies estimated the total amount of tocochromanols in *C. oxyacantha* and *C. tinctorius* to be around 57.9 and 58.2 mg/100 g oil, respectively [20].

#### 3.1.3. Carotenoids

In total, six carotenoid compounds (neoxanthin, violaxanthin, lutein, zeaxanthin, β-cryptoxanthin, and β-carotene) were identified in *C. oxyacantha* and *C. tinctorius*. β-carotene can be considered as a metabolite marker for distinguishing safflower species as the species found with a specific concentration. However, the most prevalent carotenoid compound found in safflower species is zeaxanthin, which constitutes around 37% and 58% of total carotenoids [20].

#### 3.1.4. Phytosterols

Ten and six sterols were identified in the seed oils of *C. oxyacantha* and *C. tinctorius*, respectively. Campestanol, 24-methylene cholesterol, gramisterol, 24-ethylcholest-7,24(28)dien-3beta-ol, cycloartenol, and 24-methylenecycloartanol are present in the *C. oxyacantha* species, whereas *C. tinctorius* contains avenasterol and Δ7-stigmasterol. In both species, β-sitosterol constituted the main source of phytosterols and accounted for 36.4% and 46.0% of the total amount of sterols [20].

### 3.2. Hydrophilic Compounds

#### 3.2.1. Flavonoids

Flavonoids are secondary metabolites found in the several parts of plants and mainly consist of the glycosides derived from shannesol and quercetin, safflower yellow A, hydroxysafflor yellow A, red pigment, apigenin, rutin, myricetin, carthamidin, isocarthamidin, etc. [38,39]. Flavonoids include a number of antioxidative compounds that have significant pharmacological activity. Safflower extract including flavonoids provides a protective function in the cardiac system, improving the myocardial ischemia, reducing the region of myocardial infraction, and increasing the heart rate and oxygen supply to myocardium [40]. The *C. tinctorius* extract induces adenosine diphosphate (ADP)-induced platelet aggregation and affects depolymerization of ADP in platelets. These effects can be improved by increasing the dose of safflower flavonoids [41]. The total flavones extracted from *C. tinctorius* had different hypotensive effects on the experimental animals [42].

#### 3.2.2. Saponins

Saponins are phytochemicals and natural glycosides with a wide range of pharmacological properties. The bioactive triterpenoid saponin, 3β-*O*-[β-d-xylopyranosyl(1→3)-*O*-β-d-galactopyra-nosyl]-lup-12-ene-28 oic acid-28-*O*-α-l-rhamnopyranosyl ester compound in methanolic fraction, was reported in roots of *C. tinctorius* [43]. Furthermore, recent studies reported two alkaloid–saponins compounds (*N*-coumaroylserotonin and *N*-feruloylserotonin) in *C. tinctorius* seeds [20].

### 3.3. Other Compounds

Earlier studies reported that the nutritional composition of flowers includes sugar, protein, potassium, calcium, magnesium, sodium, zinc, copper, and amino acids including aspartic acid, glutamic acid, serine, glycine, histidine, arginine, threonine, alanine, proline, tyrosine, valine methionine, cysteine, isoleucine, leucine, phenylalanine, and hydroxyl proline [44]. Safflower polysaccharide is composed of rhamnose, arabinose, xylose, mannose, glucose, galactose, and uronic acid. The lignan glyocide, tracheloside, was isolated from safflower seeds [45].

## 4. Organ-Specific Safflower Pharmacological Compounds

Safflower is a medicinal plant containing a variety of essential pharmacological compounds in nearly every part of the plant. Countries such as Pakistan and India have historically used the whole plant to increase human sexual impulses [1]. Many studies endorse safflower for cardiovascular medications, menstrual issues for women, bone pain, and swelling in trauma cases. The safflower seed contains fatty acid, vitamin E, carotenoids, flavonoids, and other compounds. Safflower seeds contain 38–48% oil, 15–22% proteins, and 11–22% fiber. The hull constitutes 18–59% of the seed weight [46]. In particular, 70% polyunsaturated linoleic acid and 10% monounsaturated oleic acid is present in the oil; linoleic acid strengthens the cells membrane using safflower oil and improves elasticity and vitality [47]. Seven antioxidative serotonin derivatives, *N*-[2- (5-hydroxy-1H-indol-3-yl)ethyl]ferulamide, *N*-[2-(5-ydroxy-1H-indol-3-yl)ethyl]-*p*-coumaramide, *N*-,*N*’-[2,2’-(5,5-dihydroxy-4,4’-bi-1H-indol-3,3’-yl)diethyl]-di-*p*-coumaramide, *N*-[2-[3’-[2-(*p*-coumaramido)ethyl]-5,5’-dihydroxy-4,4’-bi-1H-indol-3-yl]ethyl]ferulamide, *N*,*N*’-[2,2’-(5,5’-dihydroxy-4,4′-bi-1-H-indol-3,3’-yl)diethyl]-diferulamide, *N*-[2-[5-(beta-d-glucosyloxy)-1H-indol-3-yl)ethyl]-*p*-coumaramide, and *N*-[2-[5-(beta-d-glucosyloxy)-1H-indol-3-yl]-ethyl]ferulamide were isolated from the oil of safflower [48]. The main chemical compounds in the safflower petals (flower) are safflower yellow carthamidin and flavonoids. Safflower petals contain two principal pigments (yellow and red), which have been used for coloring food and textiles [49]. Recently, these pigments have been used for cosmetic coloring, such as face and hair cream, and shampoo and body lotion. In addition, the flowers have been used for important medicinal purposes such as cardiovascular, cerebrovascular, and gynecological diseases, coronary heart disease, angina pectorius, and hypertension. The safflower petals can produce a range of colors, including yellow, red, and white, based on the variety. The compounds in safflower petals are carthamin, safflower yellows A and B, safflomin A, isocarthamin, isocarthamidin, hydroxysafflor yellow A, tinctormine, puerarin, 3’-methoxyl-puerarin, and puerarinapioside pigments [50].

## 5. Safflower Pharmacological Activity

Safflower extracts from flowers and seeds, oils, and chemical components are important for the development of drugs with different pharmacological activities. The anti-nutritional factors (ANFs), called secondary metabolites, include several biologically active compounds that are distributed in oilseeds. ANFs are present in safflower in the form of tannins, luteolin, acacetin, and serotonin derivatives [51]. Safflower contains ANF compounds that can be used as anti-inflammatory, antioxidant, antibacterial, and anticoagulant agents for various health and pharmaceutical applications [52,53,54,55,56]. Previous studies of ANFs documented their ability to reduce blood glucose, plasma cholesterol, and cancer risks. However, intake of high concentrations causes adverse physiological effects [50]. The pharmacological studies of safflower extracts and the chemical components are listed in Table 1.

### 5.1. Anti-Inflammatory Effects

Several studies revealed that flower extract/compounds of hydroxysafflor yellows A and B (HSYA and HSYB) elicit various inflammatory responses, including proliferation and inflammatory responses of human fetal lung fibroblasts (MRC-5 cells) [57], inhibition of platelet activating factor (PAF)-induced proliferation, and an asthma-related inflammatory response in human bronchial smooth muscle cells (HBSMCs) [58]. In vivo studies showed that direct administration of HSYA (50, 75, and 112.5 mg/kg) to guinea pigs enhanced the protective effect on ovalbumin (OVA)-induced asthma, playing a role in controlling the asthma [59]. The flower extract, carthamin yellow (CY), reduced ischemia/reperfusion (I/R) injury in rats, aided by a reduced reactive oxygen species (ROS) release and inflammatory response [60]. The hydroxysafflor yellow B (HSYB) protected brain I/R injury through reducing the expression of inflammatory cytokines in rats [61]. HSYA attenuated lipopolysaccharide (LPS)-induced neurotoxicity and neuro-inflammation in primary mesencephalic cultures. The results suggested that HSYA has protective effects in dopaminergic neurons induced by LPS, and the mechanisms may be associated with the inhibition of inflammatory responses [62]. HSYA (14, 28, and 56 mg/kg) was intraperitoneally injected to review lipopolysaccharide (LPS)-induced acute respiratory distress syndrome in mice [63]. Safflower yellow (SY) was shown to exert an anti-inflammatory effect on BV2 microglia [64]. Safflower seed (4.3 mg/kg) compounds significantly inhibited the production of nitric oxide (NO) and pro-inflammatory cytokines, suggesting that safflower extracts produce an outstanding anti-inflammatory effect in RAW264.7 macrophages [65]. HSYA and SY inhibited myocardial apoptosis after acute myocardial infarction (AMI) and produced protective effects against myocardial ischemia in rats [66]. The therapeutic effect of HSYA on liver I/R injury was tested in vivo using a mouse model and the data suggested that HSYA can reduce I/R-induced acute liver injury by directly attenuating macrophage activation under inflammatory conditions [67]. Effect of HSYA treatment on microglia ischemia was investigated within a mouse model (BV-2 microglia cell), and the findings showed HSYA suppresses inflammatory responses induced by oxygen glucose deprivation (OGD) [68]. Neuroprotective effects of kaempferol-3-*O*-rutinoside (KRS) and kaempferol-3-*O*-glucoside (KGS) on brain injuries and the neuro-inflammatory responses in rats were reported [69]. The neuroprotective effects of HSYA administered after ischemia in the focal cerebral ischemia of rats were examined; the findings suggested that HSYA is a promising therapeutic agent for the treatment of stroke [70]. Similarly, an injection of three doses (26.7, 40, and 60 mg/kg/day) of HSYA in mice showed that HSYA attenuated the loss in body weight, increased the myeloperoxidase activity, and exerted a protective effect on the bleomycin-induced lung inflammatory response [71]. HSYA treatment (1, 5, and 10 mg/kg) showed neuro-protective qualities on rats subjected to middle cerebral artery occlusion (60 min) and reperfusion (24 h) [72]. The safflower petal aqueous extracts (SFAs) and *Carthamus* yellow (CY) reduced the LPS-induced inflammation in RAW264.7 macrophages [73]. HSYA attenuated acute lung injury (ALI) caused by the administration of LPS. In another study, male Kunming mice were pretreated with HSYA for 0.5 h prior to intraperitoneal application of LPS [74]. Effect of HSYA treatment on focal cerebral ischemia was investigated within the middle cerebral artery occlusion rat model, and the findings showed inhibitory effects on thrombosis formation and platelet aggregation [75].

### 5.2. Anti-Cancer Effects

The anti-cancer effects of HSYA were investigated in mice, where HSYA effectively blocked proliferation and migration and induced apoptosis, providing evidence as of its being an anti-cancer agent for human hepatocellular carcinoma (HCC) [76]. HSYA injected at 1.13 mg/kg in mice reduced the proportion of Tregs within the spleen and enhanced the immunity of mice, exerting an anti-cancer effect [77]. The effect of HSYB on human breast cancer MCF-7 cells showed that HSYB arrested the MCF-7 cell cycle and induced cell apoptosis [78]. Safflower seed extract treatments were orally administered 100 to 200 mg/kg weight in mice and the results showed that tumor growth decreased in cisplatin-treated mice [79]. In vitro, the activity of safflower yellow (SY) within the pulmonary metastasis of breast cancer was demonstrated, suggesting the anti-metastatic effect of SY [80]. The effects of safflower polysaccharide were examined on the proliferation and metastasis of MCF-7 human breast cancer cells; these inhibitory effects increased in a dose- and time-dependent manner [81]. The effect of HSYA on vasculogenesis in H22 tumor-bearing mice was studied and the results showed that HSYA considerably suppressed tumor growth by inhibiting secretion of angiogenesis factors, showing HSYA as a promising candidate for the prevention and treatment of HCC [82]. The teratogenic and cytotoxic effects of Carthami flos extract injected in a rat model with different dosages for zero to eight days were examined. The results showed cell differentiation [83].

### 5.3. Antioxidant Effects

The flower extract compound, *Carthamus* red, did not show any toxicity or mortality up to 2000 mg/kg doses in a rat model system; the results showed strong hepatoprotective effects and antioxidant activity in the rat model [84]. The antioxidant and anti-adipogenic effects of seed extract (CSE) were examined and the results showed that the entire phenolic and flavonoid contents of CSE were 126.0 ± 2.4 mg gallic acid equivalent (GAE)/g and 62.2 ± 1.9 mg quercetin equivalent (QE)/g, respectively. These results indicated that CSE could be a valuable source of bioactive compounds with functional food and natural antioxidant properties [85]. Safflower seed granular tea was orally administered to humans. The results showed strong antioxidant and potential bone protective effects in postmenopausal women without liver toxicity [86]. In vitro and in vivo, safflower seed extract and synthetic serotonin derivative effects showed low density lipoprotein (LDL) resistance to in vitro-induced oxidation and aortic lesion development in apoE-deficient mice. Beneficial effects were observed for preventing human cardiovascular diseases [87].

### 5.4. Vascular Effects

Rats were treated with *C. tinctorius* (CT) extract (500 mg/kg/day) for four weeks, studying renovascular hypertension. The findings suggested that CT extract produces inhibitory effects of hemodynamic alteration and vascular remodeling in 2K-1C hypertensive rats and has potent antioxidant activity [88]. HSYA was injected at different doses (0, 10, 20, and 40 mg/kg) and the effects of HSYA on hypertensive ventricular remodeling was studied using the rat model of left ventricular hypertrophy, and findings included mechanism of inhibiting cell apoptosis and suppressing metalloproteinases expression [89].

### 5.5. Osteoporosis Effects

The injection of HSYA at different concentrations (0.1, 1.0, and 10.0 μM) prevented bone mineralization, osteoblasts viability, and inhibited bone resorption. In vivo, the effect of HSYA on bone formation and glucocorticoids-induced osteoporosis (GCIOP) was demonstrated using zebrafish [90]. The effects of crude extract of safflower seed were examined on osteoblast differentiation and intracellular calcium ion concentration in MC3T3-E1 cells, showing the ability to prevent osteoporosis and protect against bone loss [91]. The effect of safflower seed oil (SSO) on osteoporosis induced-ovariectomized rats was investigated, and the findings indicate that SSO has a potential function in improving osteoporosis [92]. The effect of safflower seed extract (SSE) on periodontal tissue regeneration in a preclinical 1-wall model was evaluated in dogs and results showed improvement in bone formation [93].

### 5.6. Brain and Liver Disease Effects

The effects of safflower seed extract (100 mg kg/day) on scopolamine-induced memory impairment were tested using a mouse model. The findings suggested inhibition of cholinergic dysfunction and oxidative stress, indicating promise for memory improvement in Alzheimer’s disease (AD) patients [94]. The mechanism of safflower yellow (SY) was evaluated in amyloid β-induced AD rats, and findings revealed that SY attenuates learning and memory deficits [95]. In another study, SY improved cognitive function and astrocytes in an AD mouse model, the results showing that SY holds promise as a therapeutic approach for the treatment of AD [96]. The injection of 30 µm HSYA in rat hepatic stellate cells (HSCs) showed inhibition of HSC activation and cell proliferation, indicating it is a potential candidate for the prevention and treatment of hepatic fibrogenesis [97]. The effects of HSYA on lymphostatic encephalopathy (LE) induced brain changes in rats, supporting HSYA for the treatment of lymphostatic encephalopathy [98]. The effects of a daily dose of HSYA (5 mg/kg) to rats subjected to biweekly carbon tetrachloride (CCl_4_) injections over 12 weeks significantly reduced liver fibrosis, indicating HSYA is a promising anti-fibrotic agent for chronic liver disease [99].

### 5.7. Cell Proliferation and Inhibition Effects

The effects of HSYA were examined on transforming growth factor (TGF-β1)-induced activation of human fetal lung fibroblasts (MRC-5) [100]. HSYA affected the proliferation and adipogenesis of mouse 3T3-L1 preadipocytes [101]. The inhibitory action of HSYA on adipogenesis may occur due to the promotion of the lipolytic-specific enzyme hormone-sensitive lipase (HSL) expression by increasing HSL promoter activity [102]. The inhibitory effect of HSYA on platelet-derived growth factor (PDGF)-BB-induced vascular smooth muscle cell (VSMC) proliferation and migration was examined. The findings suggested that HSYA may be useful for the prevention and treatment of cardiovascular diseases [103]. The effect of SY on cell proliferation, migration, apoptosis, and extracellular matrix in rat aortic adventitial fibroblasts (AFs) was reported [104]. The effects of SYB on angiotensin II-induced injury to human umbilical vein endothelial cells (HUVECs) were reported [105]. The effect of *N*-(*p*-coumaroyl) serotonin (CS) and *N*-feruloylserotonin (FS) were investigated on cultured rat vascular smooth muscle cells (VSMCs), and results suggest that CS and FS contribute to vascular health and the prevention of cardiovascular disease [106]. The effect of CS on the proliferation of various cell types was examined and findings showed derivatives of CS, revealing growth-promoting activity [107].

### 5.8. Other Effects

The oil rich γ-linolenic acid was investigated for the physiological activity on mice and the results showed changes in hepatic fatty acid metabolism [108]. The effects of HSYA on hair growth cell proliferation and hair growth-associated gene expression in dermal papilla cells and keratinocytes (HaCaT) were determined in vitro and in vivo [109]. The effects of HSYA doses of 50, 100, and 200 µg on UV exposure in mice were studied, and skin damage was significantly improved [110]. The flower extract was analyzed for the treatment of diabetes and its complications. The flowers regenerated and restored Langerhans islets, thereby elevating the insulin level [111]. Subchronic toxicity of HSYA was tested on Sprague–Dawley rats using daily intraperitoneal (i.p.) injection of HSYA at doses of 180, 60, and 20 mg/kg for 90 days. The results showed slight nephrotoxicity within the rats [112]. In vitro, safflower seed extract was injected (2.1 g/day) into human subjects, with results showing oxidative insult and a pro-inflammatory response [113]. The effect of the melanogenesis inhibitory activity was evaluated as a skin whitening agent using seed compounds (*N*-feruloylserotonin, *N*-(*p*-coumaroyl) serotonin, and acacetin) [114].

## 6. Materials and Methods

This review was systemically retrieved, and analyzed the research articles, reviews, comments, and other notes reported on safflower from open access public databases including Web of Science, PubMed, Google Scholar, and Science Direct. These databases were searched with several key words related to safflower separately or combined with one another, which includes safflower, *Carthamus tinctorius*, metabolites, pharmacology studies, and antioxidant and anti-inflammatory effects. More than one hundred research papers were identified in our search and included in this review. Notably, over fifty pharmacological effects associated with safflower extracts of flower and seed, and a number of metabolites derived from safflower were described in this review. The germplasm and cultivar information of safflower was obtained from the Germplasm Resources Information Network database (https://training.ars-grin.gov/gringlobal/search.aspx).

## 7. Conclusion and Future Perspectives

The objective of this review was to focus on the pharmacological importance of the safflower species due to its diverse biologically significant metabolic profiles. Despite its several chemical constituents, such as quinochalcones, flavonoids, alkaloids, polyacetylenes, alkane-diol, fatty acids, steroids, and lignans, only a few secondary metabolites (quinochalcones and flavonoids) were investigated in detail. Single compound isolation or purification is expected to have many other potential applications in medicine, in addition to crude extracts. Hence, future studies should focus on the identification and characterization of the individual metabolites of safflower, which are essential for thorough understanding of the pharmacological significance of safflower. Quinochalcones and flavonoids are considered the characteristic and active constituents of safflower. The content variations of other primary metabolites among different regions of safflower cultivars need to be evaluated using the newly established analytical approaches. Overall, the current and emerging approaches in metabolomics applied to safflower research need to be extended and fine-tuned to improve quality and productivity. Based on current information, the pharmacological functions, including the antioxidant, anti-inflammatory, anti-diabetic, and hepatoprotective effects of safflower, can be effectively exploited in the development of new drugs to treat various human diseases.

## Figures and Tables

**Table 1:** List of safflower metabolites/extracts and their known uses in the pharmacology.

Source	Chemical Compound/Extract	Model	Effect	Reference
**Anti-Inflammatory Effect**				
Flower	Hydroxysafflor yellow A	Human (MRC-5 Cell)	Inhibits TNF-α-induced inflammation of human fetal lung fibroblasts	[57]
Flower	Hydroxysafflor yellow A	Human (bronchial smooth muscle cell)	Inhibits pro-inflammatory platelet-activating factor (PAF)-induced activation of human bronchial smooth muscle cells	[58]
Flower	Hydroxysafflor yellow A	Guinea pig	Alleviates ovalbumin-induced asthma	[59]
Flower	Carthamin yellow	Rat	Reduces ischemia-reperfusion injury	[60]
Flower	Hydroxysafflor yellow B	Rat (PC12 cell)	Protects brain against cerebral ischemia reperfusion injury	[61]
Flower	Hydroxysafflor yellow A	Mouse (primary mesencephalic cultures)	Attenuates lipopolysaccharide-induced neurotoxicity and neuroinflammation	[62]
Flower	Hydroxysafflor yellow A	Mouse	Alleviates lipopolysaccharide-induced acute respiratory distress syndrome	[63]
Flower	Safflower yellow	Mouse (BV-2 microglia cell)	Regulates microglia polarization and inhibits inflammatory response in LPS-stimulated BV2 cells	[64]
Seed	Acacetin, cosmosiin, *N*-feruloyl serotonin, and *N*-(*p*-coumaroyl) serotonin	Mouse (RAW 264.7 macrophages cell)	Anti-inflammatory effect in lipopolysaccharide-stimulated RAW 264.7 macrophages	[65]
Flower	Hydroxysafflor yellow A	Rat	Protective effects against myocardial ischemia	[66]
Flower	Hydroxysafflor yellow A	Mouse	Attenuates ischemia/reperfusion-induced liver injury	[67]
Flower	Hydroxysafflor yellow A	Mouse (BV-2 microglia cell)	Suppresses inflammatory responses of BV2 microglia	[68]
Flower	Kaempferol-3-*O*-rutinoside and Kaempferol-3-*O*-glucoside	Rat	Neuroprotective effect of kaempferol glycosides against brain injury and neuroinflammation	[69]
Flower	Hydroxysafflor yellow A	Rat	Attenuates brain ischemic injury	[70]
Flower	Hydroxysafflor yellow A	Mouse	Alleviates early inflammatory response of bleomycin-induced mice lung injury	[71]
Flower	Hydroxysafflor yellow A	Rat	Neuroprotection of hydroxysafflor yellow A in the transient focal ischemia	[72]
Flower	Safflower petal aqueous extracts & *Carthamus* yellow	Mouse (RAW264.7 cell)	Anti-inflammatory effect in lipopolysaccharide-stimulated RAW 264.7 macrophages	[73]
Flower	Hydroxysafflor yellow A	Mouse	Attenuates lipopolysaccharide-induced pulmonary inflammatory injury	[74]
Flower	Hydroxysafflor yellow A	Rat	Effects on focal cerebral ischemic injury	[75]
**Anti-Cancer Effect**				
Flower	Hydroxysafflor yellow A	Mouse & human (HepG2 cell)	Suppresses angiogenesis of hepatocellular carcinoma	[76]
Flower	Hydroxysafflor yellow A	Mouse	Anti-cancer effect in a mouse model of hepatocellular carcinoma	[77]
Flower	Hydroxysafflor yellow B	Human (breast cancer MCF-7 cell)	Effect on proliferation of cancer cells	[78]
Seed	Seed extract	Human colorectal carcinoma RKO cell and RKO-transplanted mouse	Synergizes the therapeutic effect of cisplatin and reduces cisplatin-induced nephrotoxicity	[79]
Flower	Safflower yellow	Human (COS7, MDA-MB-435s and MCF7 cell) & mouse	Prevents pulmonary metastasis of breast cancer	[80]
Flower	Safflower polysaccharide	Human (breast cancer cell, MCF-7)	Inhibits the proliferation and metastasis of breast cancer cell	[81]
Flower	Hydroxysafflor yellow A	Mouse	Inhibits angiogenesis of hepatocellular carcinoma	[82]
Flower	Carthami flos	Rat	Teratogenic and cytotoxic effect	[83]
**Antioxidant Effect**				
Flower	*Carthamus* red	Rat	Exerts antioxidant and hepatoprotective effects against CCl(4)-induced liver damage	[84]
Seed	Seed extract	Mouse (3T3-L1 cell)	Antioxidant activity and anti-adipogenic effect	[85]
Seed	Seed granular tea	Human	Antioxidant and potential bone protecting effects in postmenopausal women	[86]
Seed	Seed extract	Human	Affects markers of oxidative stress, inflammation, and aortic stiffness	[87]
**Vascular Effect**				
Flower	*Carthamus tinctorius L.* extract	Rat	Ameliorates hemodynamic alteration and vascular remodeling	[88]
Flower	Hydroxysafflor yellow A	Rat	Effects on hypertensive ventricular remodeling	[89]
**Osteoporosis Effect**				
Flower	Hydroxysafflor yellow A	Zebrafish	Promotes bone mineralization and inhibits bone resorption which reverses glucocorticoids-induced osteoporosis	[90]
Seed	Seed crude and aqueous extract	Mouse (MCT3T3-E1 cell)	Effect on osteoblast differentiation and intracellular calcium ion concentration	[91]
Seed	Seed oil	Rat	Improves osteoporosis-induced ovariectomized rats	[92]
Seed	Seed extract	Dog	Stimulates periodontal regeneration	[93]
**Brain and Liver Disease Effect**				
Seed	Seed extract	Mouse	Attenuates memory impairment induced by scopolamine	[94]
Flower	Safflower yellow	Rat	Attenuates learning and memory deficits in amyloid β-induced Alzheimer’s disease	[95]
Flower	Safflower yellow	Mouse	Neuroprotective effects on animal models of vascular dementia and Alzheimer’s diseases	[96]
Flower	Hydroxysafflor yellow A	Rat (Hepatic stellate cell)	Potential treatment for hepatic fibrogenesis	[97]
Flower	Hydroxysafflor yellow A	Rat	Attenuates lymphostatic encephalopathy-induced brain injury	[98]
Flower	Hydroxysafflor yellow A	Rat	Protects against chronic carbon tetrachloride-induced liver fibrosis	[99]
**Cell Proliferation and Inhibition Effect**				
Flower	Hydroxysafflor yellow A	Human (MRC-5 cell)	Inhibits TGF-β1-induced activation of human fetal lung fibroblasts	[100]
Flower	Hydroxysafflor yellow A	Mouse (3T3-L1 preadipocyte)	Inhibits the proliferation and differentiation of 3T3-L1 preadipocytes	[101]
Flower	Hydroxysafflor yellow A	Mouse (3T3-L1 preadipocyte)	Inhibits the proliferation and adipogenesis of 3T3-L1 preadipocytes	[102]
Flower	Hydroxysafflor yellow A	Rat	Inhibitory effects of HSYA on PDGF-BB-induced proliferation and migration of vascular smooth muscle cells	[103]
Flower	Safflower yellow	Rat	Inhibits angiotensin II-induced adventitial fibroblast proliferation and migration.	[104]
Flower	Safflower yellow B	Human (umbilical vein endothelial cell)	Protects endothelial cells from Ang II-induced cell damage	[105]
Seed	*N*-(*p*-coumaroyl) serotonin and *N*-feruloylserotonin	Rat (vascular smooth muscle cell)	Inhibits platelet-derived growth factor-BB-evoked proliferation and migration of the vascular smooth muscle cells	[106]
Seed	*N*-(*p*-Coumaroyl) serotonin	Human (lung fibroblast cell line TIG-1, MRC-5, MRC-9) and mouse (fibroblast cell line 3T3)	Grows the proliferation of normal human and mouse fibroblasts	[107]
Other Effects				
Seed	γ- Linoleic acid	Mouse	Affects hepatic fatty acid metabolism, and serum lipid levels in genetically hyperlipidemic mice deficient in apolipoprotein E expression	[108]
Flower	Floret extract	Mouse & Human (dermal papilla cells and HaCaT)	Hair growth-promoting effect	[109]
Flower	Hydroxysafflor yellow A	Mouse	Protective effect of skin photoaging induced by ultraviolet irradiation	[110]
Flower	Flower extract	Rat	Effect on treatment of diabetes	[111]
Flower	Hydroxysafflor yellow A	Rat	Subchronic toxicity of hydroxysafflor yellow A	[112]
Seed	Seed extract	Mouse	Inhibits low-density lipoprotein (LDL) oxidation and attenuate atherosclerotic lesion development	[113]
Seed	Seed extract	*Streptomyces bikiniensis* and mouse (B16 melanoma cell)	Melanogenesis inhibitory activity	[114]
